# Warming alters cascading effects of a dominant arthropod predator on fungal community composition in the Arctic

**DOI:** 10.1128/mbio.00590-24

**Published:** 2024-06-04

**Authors:** Amanda M. Koltz, Akihiro Koyama, Matthew Wallenstein

**Affiliations:** 1 Department of Integrative Biology, University of Texas at Austin, Austin, Texas, USA; 2 Department of Forestry, Michigan State University, East Lasing, Michigan, USA; 3 Department of Soil and Crop Sciences, Colorado State University, Fort Collins, Colorado, USA; Oregon State University, Corvallis, Oregon, USA; Vrije Universiteit Amsterdam, Amsterdam, the Netherlands

**Keywords:** wolf spiders, climate warming, litter microbiome, decomposition, Arctic tundra, trophic cascade

## Abstract

Rapid climate change in the Arctic is altering microbial structure and function, with important consequences for the global ecosystem. Emerging evidence suggests organisms in higher trophic levels may also influence microbial communities, but whether warming alters these effects is unclear. Wolf spiders are dominant Arctic predators whose densities are expected to increase with warming. These predators have temperature-dependent effects on decomposition via their consumption of fungal-feeding detritivores, suggesting they may indirectly affect the microbial structure as well. To address this, we used a fully factorial mesocosm experiment to test the effects of wolf spider density and warming on litter microbial structure in Arctic tundra. We deployed replicate litter bags at the surface and belowground in the organic soil profile and analyzed the litter for bacterial and fungal community structure, mass loss, and nutrient characteristics after 2 and 14 months. We found there were significant interactive effects of wolf spider density and warming on fungal but not bacterial communities. Specifically, higher wolf spider densities caused greater fungal diversity under ambient temperature but lower fungal diversity under warming at the soil surface. We also observed interactive treatment effects on fungal composition belowground. Wolf spider density influenced surface bacterial composition, but the effects did not change with warming. These findings suggest a widespread predator can have indirect, cascading effects on litter microbes and that effects on fungi specifically shift under future expected levels of warming. Overall, our study highlights that trophic interactions may play important, albeit overlooked, roles in driving microbial responses to warming in Arctic terrestrial ecosystems.

**IMPORTANCE:**

The Arctic contains nearly half of the global pool of soil organic carbon and is one of the fastest warming regions on the planet. Accelerated decomposition of soil organic carbon due to warming could cause positive feedbacks to climate change through increased greenhouse gas emissions; thus, changes in ecological dynamics in this region are of global relevance. Microbial structure is an important driver of decomposition and is affected by both abiotic and biotic conditions. Yet how activities of soil-dwelling organisms in higher trophic levels influence microbial structure and function is unclear. In this study, we demonstrate that predicted changes in abundances of a dominant predator and warming interactively affect the structure of litter-dwelling fungal communities in the Arctic. These findings suggest predators may have widespread, indirect cascading effects on microbial communities, which could influence ecosystem responses to future climate change.

## INTRODUCTION

Biodiversity and ecosystem processes are largely regulated by abiotic factors relative to biotic interactions in the Arctic due to the harsh environmental conditions (1). However, the Arctic is one of the fastest warming regions on the planet, and warming is expected to further accelerate due to increasing levels of atmospheric greenhouse gases (2–4). This continued warming may strengthen the role of biotic interactions in regulating key ecosystem processes, such as microbially mediated mineralization of soil organic carbon (C).

The northern circumpolar permafrost region, including Arctic tundra, contains approximately half of the global pool of soil organic C (5). Soil organic matter accumulation in this region is attributed to slow decomposition (6) due to low temperatures (7), poor water drainage (6), and limited nutrient availability for microbial activities (8, 9). As the Arctic warms, increased decomposition of organic matter by soil microbes could result in positive feedback to climate change (10). Understanding soil microbial responses to warming is therefore critical to assess and develop predictions of global C dynamics.

The soil microbial community can be structured by warming via three main pathways: 1) direct abiotic effects of temperature, 2) indirect biotic interactions mediated by plants, and 3) higher-level consumers. Microbial community structure may not be sensitive to warming of a few degrees of Celsius in the short term (approximately a few months), as demonstrated in lab incubation (11, 12), but see (13). On the other hand, microbial communities may be structured via indirect effects mediated by plants that can respond quickly to warming (14). For example, short-term warming stimulates plant growth, leading to increased C input to soils via litter production and root exudates (15, 16). Warming-induced changes in biotic interactions among microbes and soil fauna can also result in changes to microbial structure. Soil microbial communities and their ecosystem processes are determined, in part, through complex biotic interactions with other community members in the habitat (17). Warming has the potential to indirectly influence microbial structure (18) by altering the composition, abundances, or behavior of soil fauna (19, 20) that consume microbes. Likewise, predators that trigger trophic cascades by altering the abundances or behavior of their litter- and soil-dwelling prey could impact microbial communities (21–23). However, the extent to which predators have the potential to influence soil microbial structure—or whether warming could alter indirect predator effects on the microbial community—is understudied.

Wolf spiders are among the most widely distributed and locally abundant invertebrate predators across the Arctic (24, 25). These generalist predators primarily consume litter- and soil-dwelling prey from the fungal energy channel (26, 27). Wolf spiders have also been shown to be responsive to rapid Arctic warming (28); indeed, several lines of evidence suggest warming may cause higher wolf spider densities in some areas in the future (28, 29). Warming-associated changes in their populations could alter intraspecific competition (30) and their top–down effects on detrital food webs, with consequences for critical ecosystem processes (31). For example, previous work has shown that variation in wolf spider densities influences decomposition rates, but that effects depend upon environmental conditions (31, 32). In the Arctic, warming is associated with wolf spiders consuming a higher proportion of fungal-derived resources (27), suggesting wolf spiders could have indirect effects on fungal communities. Although microbes are susceptible to changing trophic interactions (33), whether wolf spiders play a role in structuring soil microbial communities remains an open question. Given expected increases in their densities under climate change, wolf spiders are an excellent model system to investigate how warming may alter predator-induced trophic cascades on soil microbial communities.

In this study, we investigate responses by litter-dwelling fungal and bacterial communities to expected variation in wolf spider densities and warming in an Arctic tundra ecosystem. Specifically, we used field mesocosms and open-topped warming chambers to manipulate densities of generalist-feeding wolf spider predators and ambient temperature over two summers in a well-studied area of moist acidic tundra in northern Alaska. Previous results from this experiment showed that cascading effects of wolf spiders on decomposition were different under ambient temperature vs experimental warming (31). Specifically, after 14 months of *in situ* litter incubation, higher wolf spider densities led to increased decomposition under ambient temperature but less decomposition under warming (31). Other community-level data indicated that the observed indirect effects of wolf spiders on litter decomposition were mediated by fungivorous Collembola; while more wolf spiders per plot reduced numbers of fungivorous Collembola under ambient temperature, they were associated with more Collembola under warming (31). The combined treatment effects on litter decomposition and Collembola suggest there may have been interactive effects of wolf spider densities and warming on the litter microbial communities as well. In this study, we report on responses by litter-dwelling fungal and bacterial communities after short (2-month) and longer-term (14-month) *in situ* litter incubation periods under these treatments. Based on previous findings from this system, we hypothesize that the warming and wolf spider density treatments interactively structure fungal and bacterial communities, but that effects are stronger for the fungal than bacterial communities.

## RESULTS

### Litter characteristics

**Fig 1:**
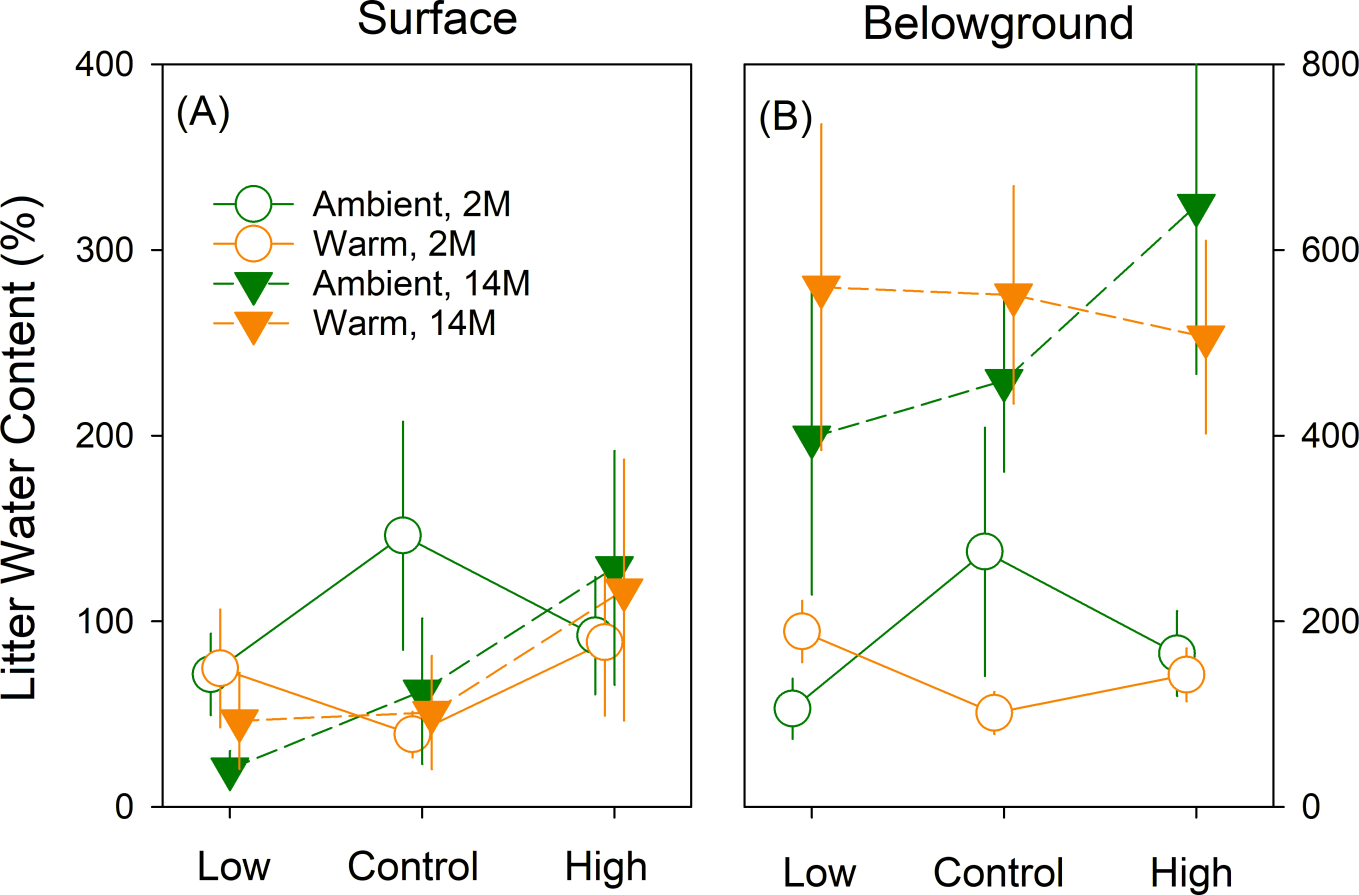
Water content of litter incubated in the field for 2 months (2011) and 14 months (2012). Results of mixed-effect ANOVAs are shown in Table 1. 2M and 14M represent 2-month and 14-month incubations, respectively. Error bars show standard errors.

There were no significant effects of wolf spider density or warming treatments on litter water content at either soil profile in either year (Fig. 1; Table 1), indicating that any potential indirect effects of experimental warming on microbial composition or decomposition due to water content were likely few. Water content in belowground litter was more than three times higher after 14- than 2-month incubation periods (*P* < 0.001, Table 1; Fig. 1B).

**TABLE 1:** Results of mixed-effect ANOVAs for three litter characteristics (water content, mass remaining, and C:N ratio)^*a*^

Profile	Predictive variables	Litter water content	Mass remaining	C:N ratio
Surface	Time	*F_1,44_* = 1.62 *P* = 0.209	*F_1,44_* = 58.30 ***P* < 0.001**	*F_1,44_* = 0.96 *P* = 0.330
	Temp	*F_1,44_* = 0.61 *P* = 0.438	*F_1,44_* = 0.64 *P* = 0.428	*F_1,44_* = 0.85 *P* = 0.360
	Spider	*F_2,44_* = 1.63 *P* = 0.207	*F_2,44_* = 1.28 *P* = 0.290	*F_2,44_* = 3.69 ***P* = 0.030**
	Time × temp	*F_1,44_* = 0.75 *P* = 0.390	*F_1,44_* = 0.65 *P* = 0.426	*F_1,44_* = 0.09 *P* = 0.760
	Time × spider	*F_2,44_* = 0.69 *P* = 0.506	*F_2,44_* = 1.58 *P* = 0.217	*F_2,44_* = 0.14 *P* = 0.870
	Temp × spider	*F_2,44_* = 0.76 *P* = 0.474	*F_2,44_* = 1.82 *P* = 0.174	*F_2,44_* = 0.23 *P* = 0.800
	Time × temp × spider	*F_2,44_* = 0.4 *P* = 0.675	*F_2,44_* = 1.90 *P* = 0.162	*F_2,44_* = 1.35 *P* = 0.270
Belowground	Time	*F_1,43_* = 59.91 ***P* < 0.001**	*F_1,43_* = 221.44 ***P* = 0.001**	*F_1,43_* = 9.63 ***P* < 0.001**
	Temp	*F_1,43_* = 0.36 *P* = 0.550	*F_1,43_* = 2.95 *P* = 0.090	*F_1,43_* = 1.17 *P* = 0.990
	Spider	*F_2,43_* = 0.61 *P* = 0.550	*F_2,43_* = 2.01 *P* = 0.150	*F_2,43_* = 1.17 *P* = 0.320
	Time × temp	*F_1,43_* = 0.04 *P* = 0.850	*F_1,43_* = 0.21 *P* = 0.650	*F_1,43_* = 1.48 *P* = 0.230
	Time × spider	*F_2,43_* = 0.53 *P* = 0.590	*F_2,43_* = 2.89 *P* = 0.070	*F_2,43_* = 0.80 *P* = 0.460
	Temp × spider	*F_2,43_* = 1.90 *P* = 0.160	*F_2,43_* = 7.12 ***P* < 0.001**	*F_2,43_* = 4.36 ***P* = 0.020**
	Time × temp × spider	*F_2,43_* = 0.73 *P* = 0.490	*F_2,43_* = 4.10 ***P* = 0.020**	*F_2,43_* = 2.38 *P* = 0.100

^*a*^ Significant effects (i.e., *P* < 0.05) are in bold.

**Fig 2:**
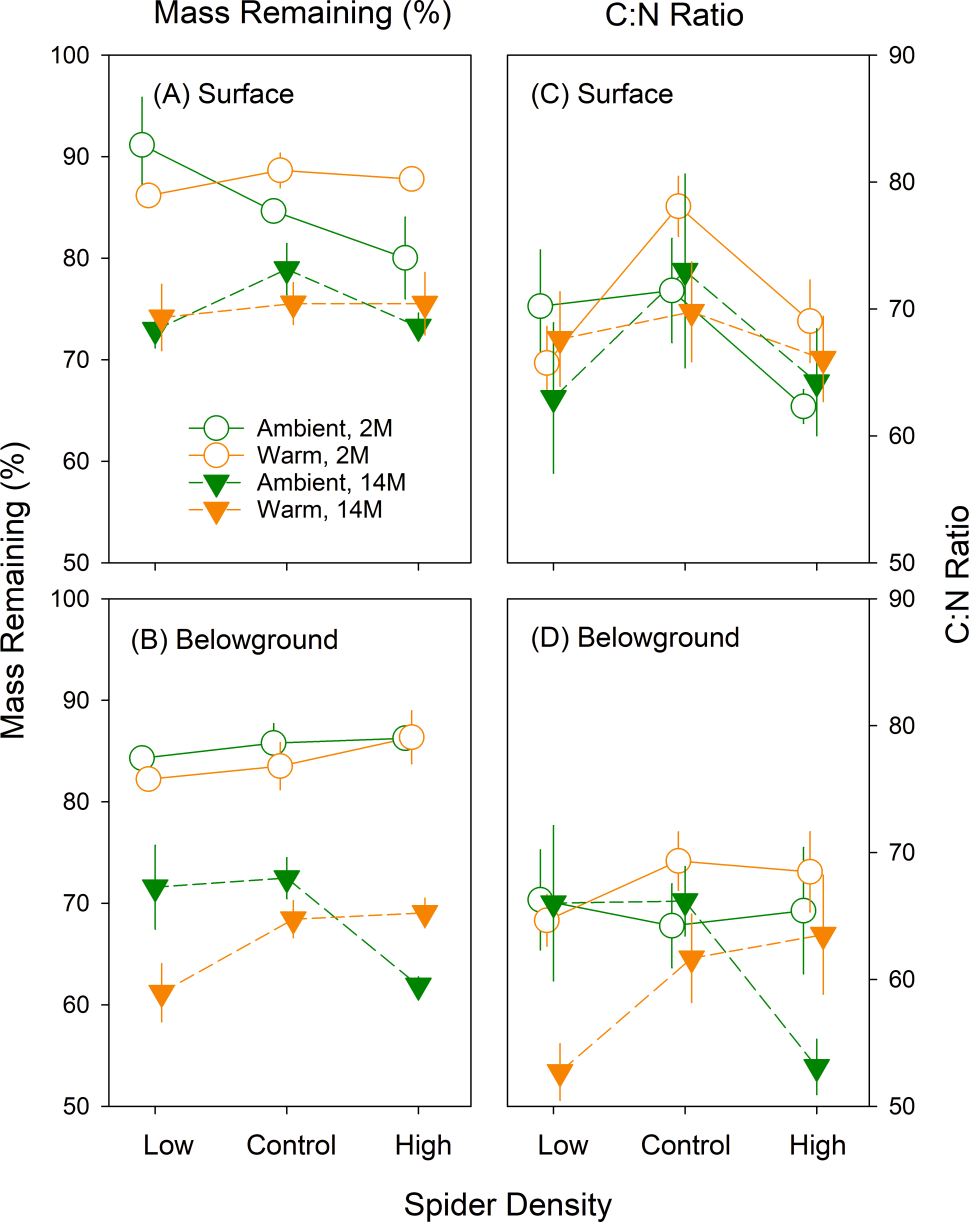
Remaining mass and C:N ratio of *Eriophorum vaginatum* litter recovered after 2 (2011) and 14 months (2012) of incubations in the field. Results of mixed-effect ANOVAs are shown in Table 1. Error bars show standard errors.

Litter mass loss occurred due to decomposition at both the surface and belowground during the course of the experiment (*P* < 0.001, Table 1; Fig. 2A and B; Fig. S1A and B). After 2-month incubation, surface and belowground litter lost, on average, 14% and 15%, respectively (Fig. 2A and B; Fig. S1A and B). After 14-month incubation, less belowground litter than surface litter remained (32.6% vs 24.9% on average, Fig. 2A and B; Fig. S1A and B). As previously reported (31), there were significant interactive effects of warming and wolf spider density treatments on belowground litter mass loss, which were dependent on incubation periods (*P* = 0.020, Table 1; Fig. 2B; Fig. S1B). Although the treatments did not affect belowground mass loss after 2-month incubation, there were significant interactive treatment effects on belowground mass loss after 14-month incubation (Fig. 2B; Fig. S1B; Table S1). Specifically, under ambient temperature, belowground litter in the high wolf spider density treatment lost approximately 10% more mass than litter in the low or control wolf spider density treatments (Fig. 2B; Fig. S1B), whereas under warming, litter in low wolf spider density plots lost approximately 8% more mass than those in control and high wolf spider density plots (Fig. 2B; Fig. S1B). Neither warming nor wolf spider density treatment significantly affected litter mass loss at the soil surface after two or 14 months (Fig. 2A; Table 1; Fig. S1A; Table S1).

In terms of litter nutrient content, there was no significant change in the surface litter C to nitrogen (N) ratio (C:N ratio) between 2- and 14-month incubation (Table 1;Fig. 2C; Fig. S1C). However, higher wolf spider densities were associated with a lower surface litter C:N ratio within each temperature treatment (*P* = 0.030, Table 1; Fig. 2C; Fig.S1C), and this trend was more apparent after 2- than 14-month incubation (Fig. 2C; Table S1). For the belowground litter, the C:N ratio declined over time between 2- and 14-month incubation (*P* < 0.001, Table 1; Fig. 2D; Fig. S1D). Additionally, there were significant interactive effects of the wolf spider density and warming treatments on the belowground litter C:N ratio (*P* = 0.020, Table 1; Fig. 2D; Fig. S1D), which were driven by the C:N ratio of belowground litter after 14-month incubation (*P* = 0.019, Table S1; Fig. 2D; Fig. S1D), similar to described patterns in decomposition (Fig. 2B; Fig. S1B). Specifically, under ambient temperature, the belowground litter C:N ratio was 20% lower under high wolf spider density than low and control wolf spider densities (Fig. 2D; Fig. S1D). However, under experimental warming, the belowground litter C:N ratio was 16% lower under the low wolf spider density treatment than under control and high wolf spider densities (Fig. 2D; Fig. S1D).

### Alpha diversity of bacteria and fungi

**Fig 3:**
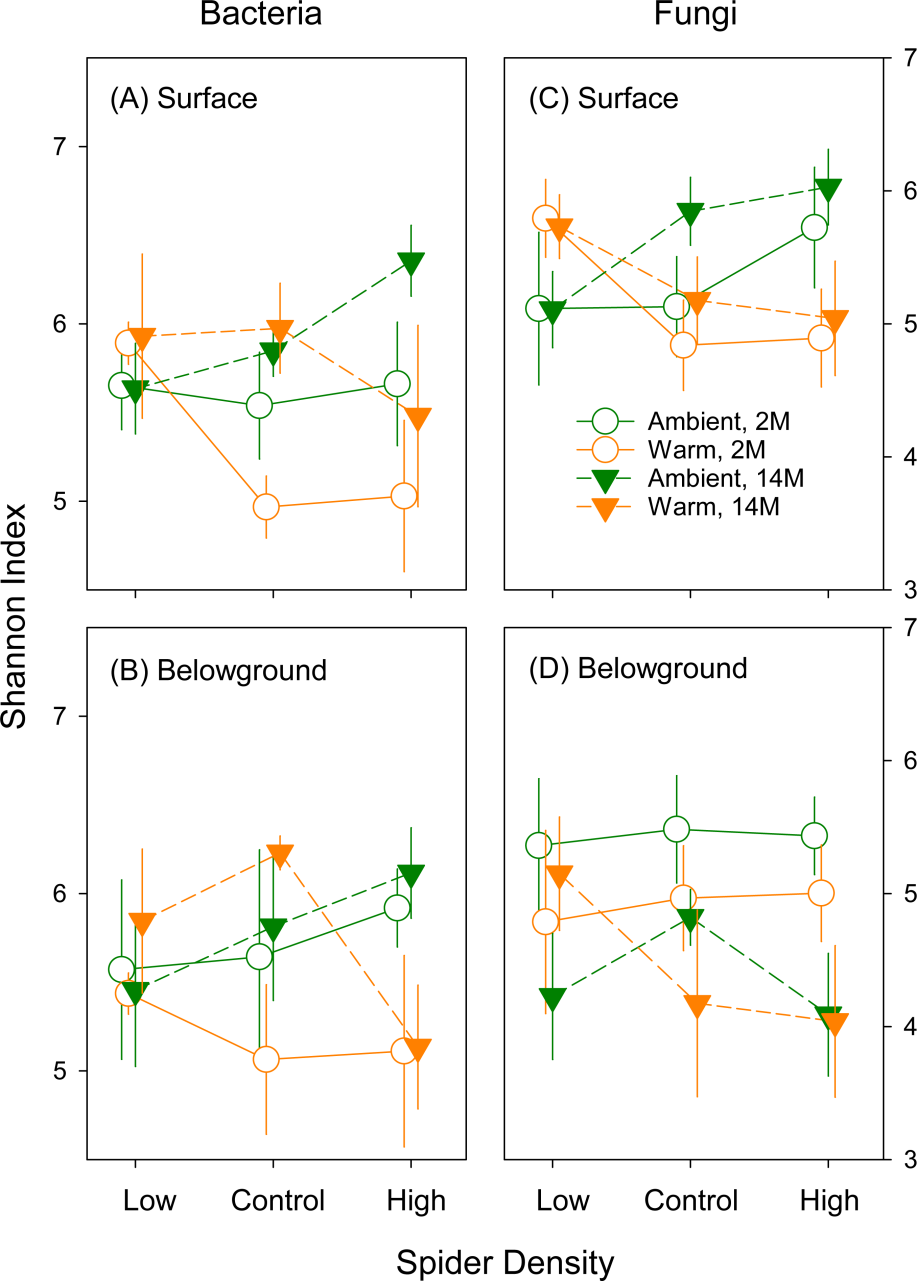
Shannon diversity indices of bacterial and fungal communities in the surface and belowground litter collected after 2-month (2011) and 14-month (2012) incubations. Results of mixed-effect ANOVAs are shown in Table 2. 2M and 14M represent 2-month and 14-month incubations, respectively. Error bars show standard errors.

Bacterial alpha diversity in surface litter as assessed via Shannon indices was higher after 14- than 2-month incubation (*P* = 0.032, Table 2; Fig. 3A). No temporal trend in bacterial diversity was found in belowground litter (Table 2; Fig. 3B). Likewise, there were no significant effects of the warming or wolf spider density treatment on bacterial diversity in surface or belowground litter (Table 2; Fig. 3A and B). Bacterial richness (i.e., observed OTUs) showed similar trends as bacterial Shannon diversity indices (Fig. S2A and B; Table S2). The bacterial richness and Shannon diversity indices were tightly positively correlated in each profile (Fig. S3A and B), indicating that richness, as well as evenness, contributed to the bacterial Shannon diversity indices.

**TABLE 2:** Results of mixed-effect ANOVAs for Shannon diversity indices^*a*^

Predictive variables	Bacteria		Fungi	
	Surface	Belowground	Surface	Belowground
Time	*F_1,38_* = 4.97 ***P* = 0.032**	*F_1,39_* = 1.76 *P* = 0.192	*F_1,38_* = 1.12 *P* = 0.297	*F_1,41_* = 7.48 ***P* = 0.009**
Temp	*F_1,38_* = 2.42 *P* = 0.128	*F_1,39_* = 1.41 *P* = 0.243	*F_1,38_* = 1.74 *P* = 0.195	*F_1,41_* = 0.78 *P* = 0.381
Spider	*F_2,38_* = 0.79 *P* = 0.463	*F_2,39_* = 0.15 *P* = 0.860	*F_2,38_* = 0.33 *P* = 0.718	*F_2,41_* = 0.19 *P* = 0.826
Time × temp	*F_1,38_* = 0.09 *P* = 0.766	*F_1,39_* = 1.10 *P* = 0.300	*F_1,38_* = 0.57 *P* = 0.455	*F_1,41_* = 1.11 *P* = 0.299
Time × spider	*F_2,38_* = 1.13 *P* = 0.333	*F_2,39_* = 0.72 *P* = 0.493	*F_2,38_* = 0.57 *P* = 0.572	*F_2,41_* = 0.56 *P* = 0.573
Temp × spider	*F_2,38_* = 2.43 *P* = 0.102	*F_2,39_* = 1.59 *P* = 0.217	*F_2,38_* = 3.87 ***P* = 0.030**	*F_2,41_* = 0.66 *P* = 0.523
Time × temp × spider	*F_2,38_* = 0.65 *P* = 0.530	*F_2,39_* = 0.53 *P* = 0.594	*F_2,38_* = 0.04 *P* = 0.963	*F_2,41_* = 0.84 *P* = 0.438

^*a*^ Significant effects (i.e., *P* < 0.05) are in bold.

Warming and wolf spider densities had significant interactive effects on fungal diversity in surface litter (*P* = 0.030, Table 2). After both 2- and 14-month incubation, higher wolf spider densities were associated with higher fungal diversity under ambient temperature but lower fungal diversity under warming (Fig. 3C). For belowground litter, overall fungal diversity was higher after 2- than 14-month incubation (*P* = 0.009, Table 2; Fig. 3D) but was not significantly affected by the experimental treatments (Table 2). Fungal richness also showed similar trends as fungal Shannon diversity indices (Fig. S2C and D; Table S2). Even though the fungal richness and Shannon diversity indices were significantly correlated, the R^2^ values were lower than those for bacterial communities, especially for the surface profile (Fig. S3), indicating greater evenness contributions to fungal than bacterial Shannon diversity indices.

### Temporal beta diversity of bacteria and fungi

**Fig 4:**
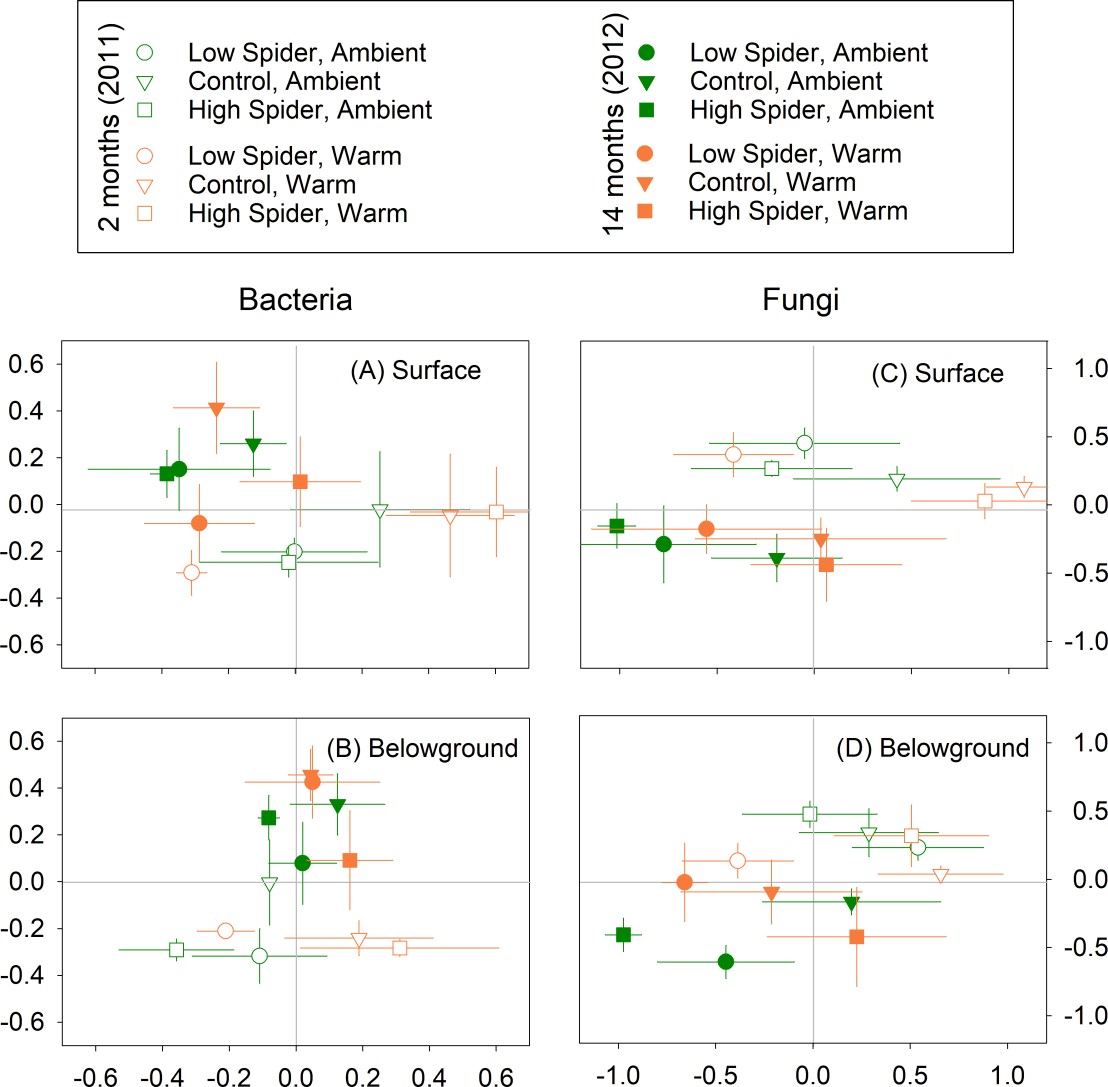
Results of non-metric multidimensional scaling (NMDS) for bacterial and fungal communities at the OTU level in surface and belowground litter collected after -month (2011) and 14-month (2012) incubations. NMDS was conducted for each microbial group and soil profile. Results of PERMANOVA are shown in Table 3. Error bars show standard errors.

Bacterial community structure at the OTU level was different between litter incubated for 2 months vs 14 months at both the surface (*P* = 0.002, Table 3; Fig. 4A) and belowground (*P* = 0.001, Table 3; Fig. 4B). The wolf spider density treatment significantly altered litter bacterial community composition at the soil surface (*P* = 0.025, Table 3; Fig. 4A). When the same analyses were conducted for the bacterial community composition at the class level (Fig. S4 and S5), the wolf spider density treatment (*P* = 0.016) and the interaction between incubation times and warming were significant at the soil surface and belowground (*P* = 0.036) (Table S3).

**TABLE 3:** Results of PERMANOVA for NMDS scores at the OTU level^*a*^

Predictive variables	Bacteria		Fungi	
	Surface	Belowground	Surface	Belowground
Time	*F_1,53_* = 10.38 ***P* = 0.002** *R^2^* = 0.11	*F_1,54_* = 10.38 ***P* = 0.001** *R^2^* = 0.16	*F_1,53_* = 10.36 ***P* = 0.004** *R^2^* = 0.15	*F_1,56_* = 11.26 ***P* = 0.001** *R^2^* = 0.16
Temp	*F_1,53_* = 1.43 *P* = 0.390 *R^2^* = 0.02	*F_1,54_* = 1.43 *P* = 0.274 *R^2^* = 0.02	*F_1,53_* = 3.55 *P* = 0.061 *R^2^* = 0.05	*F_1,56_* = 0.08 *P* = 0.907 *R^2^* <0.01
Spider	*F_2,53_* = 1.32 ***P* = 0.025** *R^2^* = 0.09	*F_2,54_* = 1.32 *P* = 0.293 *R^2^* = 0.04	*F_2,53_* = 3.49 ***P* = 0.034** *R^2^* = 0.10	*F_2,56_* = 1.48 *P* = 0.231 *R^2^* = 0.04
Time × temp	*F_1,53_* = 1.2 *P* = 0.727 *R^2^* = 0.01	*F_1,54_* = 1.20 *P* = 0.289 *R^2^* = 0.02	*F_1,53_* = 0.07 *P* = 0.911 *R^2^* <0.01	*F_1,56_* = 0.84 *P* = 0.438 *R^2^* = 0.01
Time × spider	*F_2,53_* = 0.12 *P* = 0.754 *R^2^* = 0.02	*F_2,54_* = 0.12 *P* = 0.977 *R^2^* <0.01	*F_2,53_* = 0.24 *P* = 0.897 *R^2^* = 0.01	*F_2,56_* = 0.41 *P* = 0.778 *R^2^* = 0.01
Temp × spider	*F_2,53_* = 1.40 *P* = 0.140 *R^2^* = 0.06	*F_2,54_* = 1.40 *P* = 0.239 *R^2^* = 0.04	*F_2,53_* = 1.95 *P* = 0.146 *R^2^* = 0.06	*F_2,56_* = 3.27 ***P* = 0.037** *R^2^* = 0.09
Time × temp × spider	*F_2,53_* = 0.95 *P* = 0.640 *R^2^* = 0.02	*F_2,54_* = 0.95 *P* = 0.443 *R^2^* = 0.03	*F_2,53_* = 0.32 *P* = 0.796 *R^2^* = 0.01	*F_2,56_* = 1.26 *P* = 0.287 *R^2^* = 0.04

^*a*^ Significant effects (i.e., *P* < 0.05) are in bold.

Similar to the bacterial litter communities, fungal community composition at the OTU level was different between the two incubation periods in both soil profile locations (*P* ≤ 0.004, Table 3; Fig. 4CD). Likewise, wolf spider densities affected fungal community composition in surface litter (*P* = 0.034, Table 3; Fig. 4C). Lastly, there were significant interactive effects of the warming and wolf spider density treatments on fungal community composition in belowground litter (*P* = 0.037, Table 3; Fig. 4D). Results of the same analyses at the fungal class level (Fig. S5 and S6) were similar to those at the OTU level, including significant interactive treatment effects (*P* = 0.037, Table S3).

## DISCUSSION

### Interactive effects of wolf spiders and warming on litter fungal communities

We found support for our hypotheses that variation in wolf spider densities not only drives changes in litter-dwelling fungal communities but also that warming alters these effects. Previous work has shown warming (e.g., reference 34) and predator abundances (e.g., reference 18) can independently structure the communities of soil microbes, including fungi. Effects of warming on microbial communities have also been observed in multiple different terrestrial ecosystems, including Arctic tundra (35, 36), deciduous forests (37, 38), alpine tundra and meadow (39, 40), and grasslands (41–43). However, to our knowledge, the potential for predators and warming to interactively impact microbial structure and function has not been documented before. Notably, we observed effects on fungal communities at soil profile depths below which wolf spiders are typically active, suggesting the spatial scale at which wolf spiders influence ecosystem structure and function extends beyond their own microhabitat. Taken together, our findings demonstrate that abiotic conditions and biotic interactions across trophic levels interactively contribute to the structure and function of microbial communities in Arctic tundra. These results point to an underappreciated pathway by which warming influences microbial communities, which could have important implications for predicting the future of a large quantity of mineralizable soil organic C pools in the rapidly warming Arctic (44, 45).

**Fig 5:**
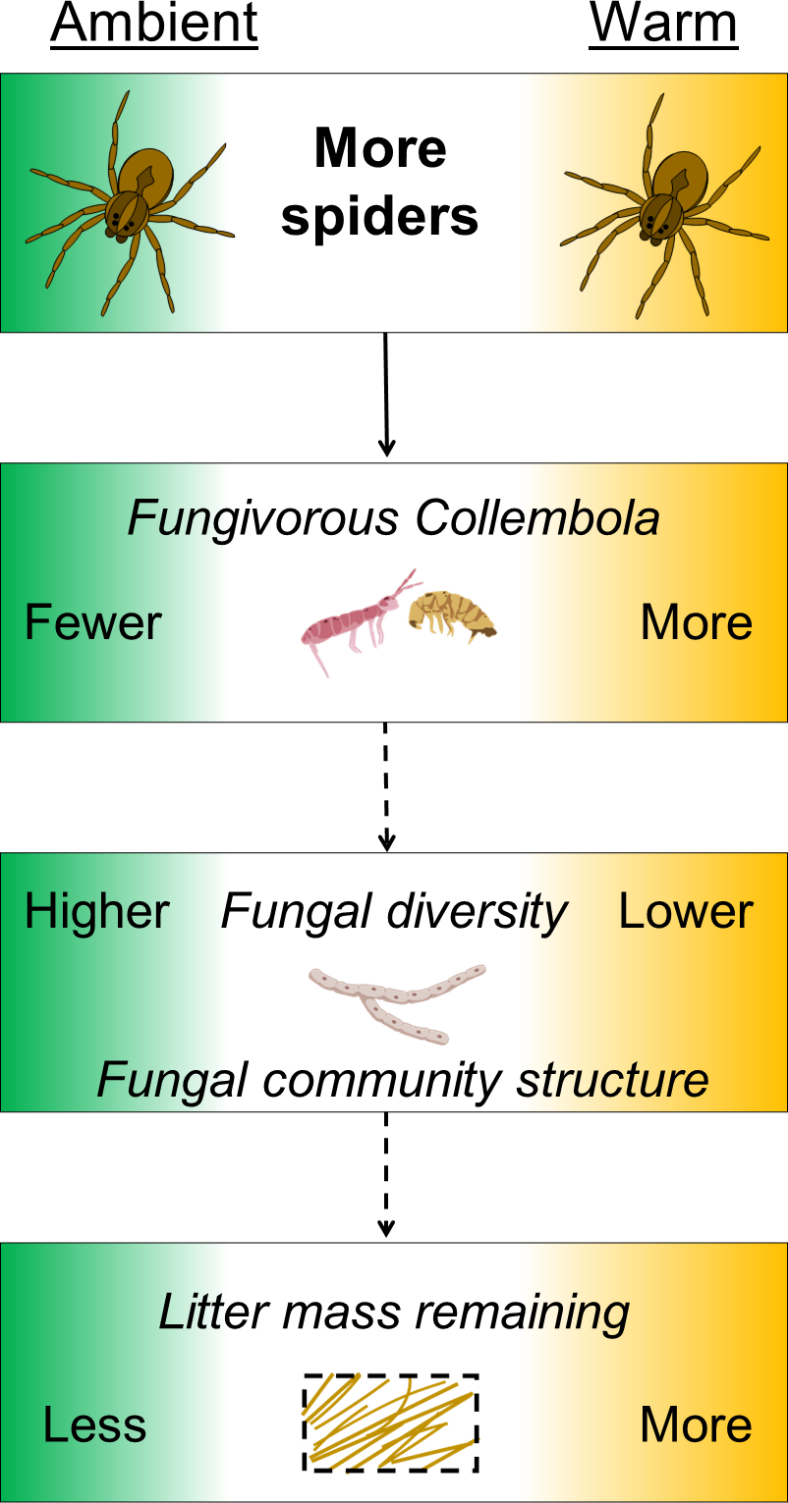
Hypothesized cascading effects of wolf spiders on fungivorous Collembola prey, the fungal community, and litter decomposition under ambient vs experimental warming from Koltz et al. (31) and this study. The solid arrow shows the direct effect of high wolf spider density on fungivorous Collembola (31). The dotted arrows are hypothesized effects based on this study: Fungivorous Collembola densities affect fungal diversity and community structure, which ultimately affects ecosystem functioning via litter decomposition.

One explanation for the interactive effects of wolf spider densities and warming on fungal communities could be a cascading effect associated with changes in consumptive pressure by fungivorous Collembola (Fig. 5). Previously published findings from this experiment demonstrated interactive effects of wolf spiders and warming on lower trophic levels, whereby higher densities of wolf spiders resulted in fewer of their Collembola prey under ambient temperature but more Collembola under warming (Fig. 5) (31). There are multiple potential mechanisms behind these interactive effects, including the possibility of intraguild predation among the spider community reducing the strength of top–down predator effects (31). Fungivorous Collembola also influence fungal community composition (46, 47) in a variety of ways, including through preferential grazing on particular fungal taxa (48, 49) and by stimulating fungal growth (50). Thus, it seems likely that some of the observed changes in the fungal communities were driven by treatment-associated variation in fungivorous Collembola abundances and grazing pressure.

In contrast, the only changes to the bacterial community occurred on surface litter in response to the wolf spider density treatment. Shifting species interactions among the litter-dwelling animal communities driven by changing wolf spider densities may have contributed to the observed changes in the surface litter bacterial communities. The interactions responsible for altering the bacterial community are unresolved, but there are numerous potential ways by which wolf spiders could directly or indirectly influence the bacterial portion of the food web. For example, spiders have been shown to affect abundances of bacterivorous nematodes and protozoa through trophic cascades (18). More generally, wolf spiders are widely acknowledged as having top–down effects on detritivores in a variety of terrestrial ecosystems (51, 52). Future research could expand upon these findings by elucidating the mechanisms through which these and other common predators influence microbes, including bacteria.

There are several ways through which warming may have influenced fungal community diversity and structure in this experiment: 1) direct abiotic effects of warming and/or indirect effects of warming mediated by 2) plants and 3) trophic interactions. Although the relative contributions of these pathways are unknown, our findings and those from other studies suggest that the direct effects of warming on fungal composition in the short term can be small. For example, results from two recent laboratory-based incubation experiments using soils collected from the Arctic tundra showed that fungal and bacterial community structure was resistant to warming across multiple temperature treatments (11, 12). An Arctic field experiment showed that increased soil temperature during winter when plants and many arthropods are dormant causes little changes in soil fungal and bacterial community compositions for the first 1.5 years (36). Any potential plant-mediated biotic effects on microbial structure were also likely limited by our experimental setup; surface litter had had little to no contact with plants, and the small mesh size on the litter bags reduced root–microbial interactions. Therefore, the findings from this experiment indicate that the effects of short-term warming on litter microbial structure were mediated by the indirect effects of wolf spiders and other biotic interactions within the detrital community, such as fungivorous Collembola (Fig. 5).

### Covariation between litter fungal communities and litter decomposition

In addition to the interactive effect of wolf spider densities and warming on litter fungal communities, we also documented the interactive effects of our experimental treatments on litter mass loss (Fig. 5). It is not possible to assess the extent to which observed fungal community compositions uniquely contributed to litter mass loss across the different treatments. Complications in addressing this question are due to the temporal-scale differences of sampling; while the microbial community compositions represent snap shots in time, litter mass loss was the result of cumulative microbial activity over the duration of the experiment. Nevertheless, studies have demonstrated that microbial structure can influence organic matter decomposition rates: Several controlled lab experiments have shown that manipulated microbial community compositions resulted in different decomposition rates (53–56). For instance, using reciprocal field transplants of inoculated litter across five ecosystems along precipitation and temperature gradients, Glassman et al. (57) demonstrated that abiotic environmental factors were a major predictor of litter decomposition rates but that the composition of the inoculated microbial community and interactions with associated environmental factors also played a role. Thus, it is plausible that the treatment-driven changes in the fungal composition and structure contributed to the observed litter decomposition rates (Fig. 5).

### Conclusion

We found that variation in wolf spider densities and warming interactively structured litter fungal community composition and modified litter decomposition rates. In polar regions where environmental conditions can be incredibly harsh, abiotic factors are considered to be the primary drivers of biodiversity and ecosystem processes (58). However, as shown here, widespread invertebrate predators can indirectly alter the effects of warming on microbial structure and key ecological processes. Given existing uncertainties around the fate of soil organic C in the Arctic, the role of predation and other types of biotic interactions in driving ecosystem structure and function warrants further attention as this region continues to warm.

## MATERIALS AND METHODS

### Experimental design

A fully factorial mesocosm field experiment was set up to explore the effects of wolf spider densities and warming on microbial community composition and litter decomposition, as described in Koltz et al. (31). The experiment was conducted from early June 2011 through late July 2012 near Toolik Field Station (68°38’N and 149°43’W, elevation 760 m) in a well-studied area of moist acidic tundra, which is the dominant tundra type on the North Slope of Alaska. The average annual temperature is −10°C, with positive temperatures occurring mainly only during the summer months, and the annual precipitation is 200 to 400 mm (59).

A total of thirty plots were randomly assigned to one of six wolf spider density/warming treatments, distributed among five blocks. Half of the plots were warmed using 1.5 meter-diameter ITEX (International Tundra Experiment) open-topped passive warming chambers, which increase the mean air temperature by 1 to 2°C (60). The warming chambers were placed over the plots during June and July of each study year only to avoid affecting snow dynamics. The wolf spider density treatments included the following: (i) low wolf spider density; (ii) control spider density, and (iii) enriched wolf spider density. In early June of each summer after snowmelt, we used live pitfall traps to remove all possible spiders from the low wolf spider density plots. Enriched plots received additional spiders collected from a nearby area to bring densities to approximately double the early season average density of the control plots. Plots were monitored with live pitfall traps periodically throughout the summer to check densities and either remove or add wolf spiders to maintain the pre-assigned density treatments. The efficacy of the wolf spider density treatments (i.e., low, control, and high densities) were verified in late summer during the week of litter bag collection (see reference 31). In 2011, estimations via 24-hour live pitfall trapping between July 20 and 22 indicated there were 0.2 (±0.10), 0.2 (±0.10), and 0.8 (±0.22) spiders per square meter (standard error) in the low, control, and high spider density treatments, respectively, at that time (31). Pitfall traps only catch a subset of individuals present, so at the end of the summer in 2012, we supplemented live pitfalls with visual surveys to more fully account for all spiders within the plots; this sampling revealed there were 0.3 (±0.21), 1.8 (±0.20), and 3.3 (±0.47) spiders per square meter in the low, control, and high spider density treatments, respectively (31). The wolf spider community in the moist acidic tundra habitat near Toolik Lake where we conducted our experiment is dominated by a single species, *Pardosa lapponica* (25). We validated this through a field survey of wolf spiders during the summer of 2012 in a similar habitat near our experimental plots, which confirmed that >95% of wolf spiders were *P. lapponica* (30).

### Moisture availability

Experimental warming, including through the use of open-topped warming chambers used here, can reduce soil moisture (61), with consequences for microbial community composition (e.g., reference 43) and litter decomposition (e.g., references 62, 63). To account for this, we measured soil moisture in three locations in each plot at the beginning, middle, and end of the 2012 summer season using a HydroSense portable soil moisture probe (Campbell Scientific, Logan, UT, USA). Soil moisture data indicated that the warming treatments did not alter average soil moisture content in our experimental plots (*P* = 0.501).

### Litter incubation

Litter bags were used to measure the response of the microbial community to variation in wolf spider densities and to warming. The litter bags were 8 cm by 8 cm with 3 mm mesh size on the top and bottom to allow access by most arthropods (other than wolf spiders and beetles). The bags were filled with 1.5 g of standing dead leaves of the dominant plant, *Eriophorum vaginatum,* which were collected during the previous summer from an area adjacent to our experimental plots, dried at 40°C for 48 hours, mixed, and sub-sampled for litter bag preparation (see reference 31). Total C and N contents were measured for ground subsamples of the initial litter mixture using a CE Elantech Flash EA 1112 Elemental Analyzer (CE Elantech, Inc., Lakewood, NJ, USA) at Duke University, Durham, NC, USA.

Two pairs of these litter bags were deployed in each experimental plot during mid-June, as described in Koltz et al. (31). From each of these pairs, one litter bag was placed on the soil surface and the other was buried in the litter layer below the moss surface (ca. 5 to 10 cm belowground). One pair of litter bags (i.e., one litter bag from the surface and one from the litter layer) ws collected after 2-month incubation and the other pair after 14-month incubation. Upon collection, accumulated soil, ingrown moss and roots, and microarthropods were manually removed from each bag containing decomposed litter, and a subsample (0.25 g) of litter was stored at −80°C for DNA extraction at a later date. The remainder of the litter was dried at 40°C for 72 hours to determine litter moisture content and proportional mass loss from the initial litter. Subsamples of dried litter were then ground and analyzed for C and N contents, as described previously.

### DNA extraction, sequencing of fungal and bacterial communities, and sequence data processing

Genomic DNA was extracted from 0.25 g sub-samples of homogenized litter from each collected litter bag using the MoBio PowerSoil DNA extraction kit (MO BIO Laboratories, Inc., Carlsbad, CA, USA), and eluted genomic DNA samples were stored at −80°C before downstream processing. The 16S and fungal ITS rRNA genes were amplified for each sample using primer sets of F515F/R806 (64) and ITS1f/ITS2 (65), respectively, which were modified for the Illumina MySeq platform (66).

Polymerase chain reactions (PCRs) were performed using triplicate 25-µL assays. Each assay consisted of 12.5 µL of KAPA2G Fast Multiplex Mix (Kapa Biosystems, Woburn, MA, USA), 0.1 µL of BSA (10.0 ng µL^−1^), 1.25 µL of each primer (10.0 µM), and 9.9 µL of a genomic DNA template (1 ng µL^−1^). The PCR thermal cycling steps consisted of an initial denaturation and enzyme activation step of 95°C for 3 min, followed by 30 cycles of 95°C for 10 sec, 50°C for 10 sec, and 72°C for 1 sec. After qualities of PCR products, including amplification and lengths, were assessed by agarose gel electrophoresis, the products were purified using the UltraClean PCR Clean-UP Kit (MO BIO Laboratories, Inc., Carlsbad, CA, USA) and quantified using the Quant-iT PicoGreen dsDNA Assay Kit (Invitrogen, Molecular Probes, Inc., Eugene, OR, USA). An equal quantity of amplicon from each sample was pooled for each of the 16S and fungal ITS PCR products. Each of the pooled amplicons were sequenced with a single run of the 2,250-bp V2 500-cycle kit on an Illumina MiSeq instrument with at Research Technology Support Facility, Michigan State University, East Lansing, MI, USA. All the sequences were deposited at GenBank of the National Center for Biotechnology Information (BioProject ID: PRJNA565353).

Bacterial 16S and fungal ITS Illumina amplicon sequences were processed via the QIIME 1.9.1 toolkit (67). For the fungal ITS sequences, only reverse reads were used for subsequent analyses because some forward reads had poor sequence quality, which would result in substantial reduction in the sequence number per sample in rarefaction. Chimeric sequences in the sequences were identified using USEARCH (68); for the 16S and ITS sequences, the reference-based method with the Greengenes database (version 13.8) (69) and the abundance-based method were used, respectively. The chimeric sequences were removed for the downstream analyses. For the 16S and IST sequences, operational taxonomic units (OTUs) were determined at the 97% similarity level (70) via USEARCH (68) using the Greengenes (13_8 version) (69) and UNITE database (Version 7) (71), respectively. All the non-bacterial sequences and singletons were removed and rarefied at 23,455 sequences per sample. The remaining sequences were aligned via PyNAST (72), and a bacterial phylogenetic tree was built using FastTree (73).

### Statistical analyses

Linear mixed-effects models were used to test the potential interactive effects of our treatments (wolf spider densities × warming) and time (i.e., 2 months and 14 months in 2011 and 2012, respectively) on the alpha diversity (Shannon index) of the fungal and bacterial communities for each soil profile. All three factors were treated as categorical variables. The interaction among spider densities, warming, and time of the litter bag collection were included as fixed effects in the models; experimental blocks were included as a random effect. Treatment and time effects were estimated separately for fungi and bacteria for each soil profile. Using the same model structure, the effects of the treatments and time on the water and nutrient content (C and N) of the litter within the litter bags were also considered. All analyses were conducted using the lme function of the nlme package (74) in R 3.5.2 (75).

In addition, variation in bacterial and fungal community composition (i.e., beta diversity) from each soil profile was assessed using non-metric multidimensional scaling (NMDS; Kruskal 1964) using the metaMDS function in the vegan package (76) in R. Each model employed two dimensions (*k* = 2) and had an acceptable stress value of <0.2 (77) (stress = 0.14 and 0.17 for bacteria from surface and belowground, respectively, and 0.11 and 0.12 for fungi in surface and belowground, respectively). To assess the effects of the spider density, warming treatments, and time of litterbag collection on microbial composition, permutational multivariate ANOVAs (PERMANOVAs) (78) were performed using the NMDS scores for each microbial group and soil profile with the adonis function in the vegan package (76). For all models, the spider density treatment was treated as a categorical variable (low, control, and high density), and treatment blocks were included as a random effect. All data were archived through the Arctic LTER Data Catalog (http://arc-lter.ecosystems.mbl.edu/data-catalog).

## Data Availability

Sequences were deposited at GenBank of the National Center for Biotechnology Information (BioProject ID: PRJNA565353).

## SUPPLEMENTAL MATERIAL

Supporting Information mbio.00590-24-s0001.pdf Fig. S1 to S6; Table S1 to S3.

ASM does not own the copyrights to Supplemental Material that may be linked to, or accessed through, an article. The authors have granted ASM a non-exclusive, world-wide license to publish the Supplemental Material files. Please contact the corresponding author directly for reuse.
