# Supplementary material for: Warming alters cascading effects of a dominant arthropod predator on fungal community composition in the Arctic
Source: mBio. 2024 Jun 4;15(7):e00590-24. doi: 10.1128/mbio.00590-24 (PMC11253614; doi:10.1128/mbio.00590-24)
Supplement: Supporting Information — Fig. S1 to S6; Table S1 to S3. [file mbio.00590-24-s0001.pdf]

Supporting Information

Authors: Amanda M. Koltz, Akihiro Koyama and Matthew Wallenstein.

Title: Warming alters cascading effects of a dominant arthropod predator on fungal community composition in the Arctic

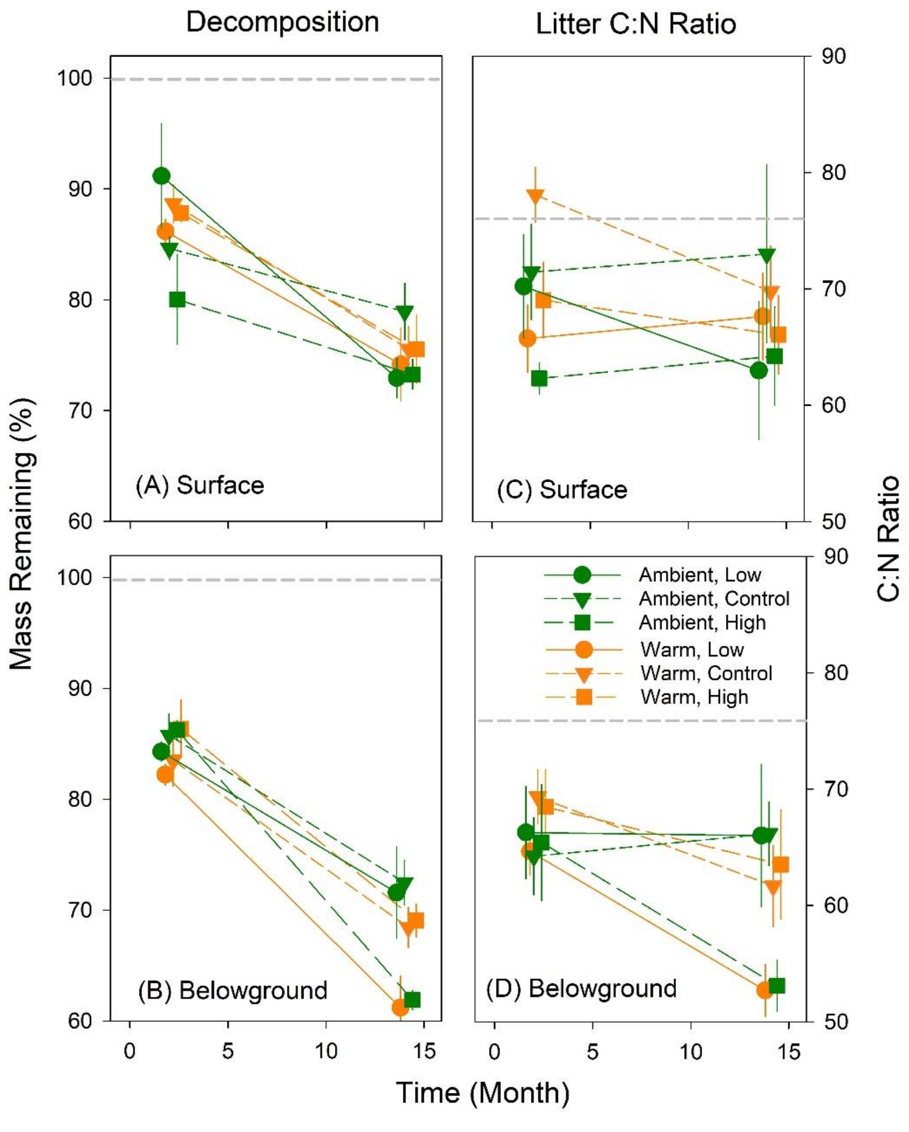

Fig. S1. Remaining mass and C:N ratio of *Eriophorum vaginatum* litter recovered after two- (2011) and 14-month (2012) incubations in the field. The same data in Fig. 2 are rearranged to show changes during the litter incubation in the field. Error bars show standard errors. The initial values of litter mass and C:N ratio are shown in gray dashed lines.

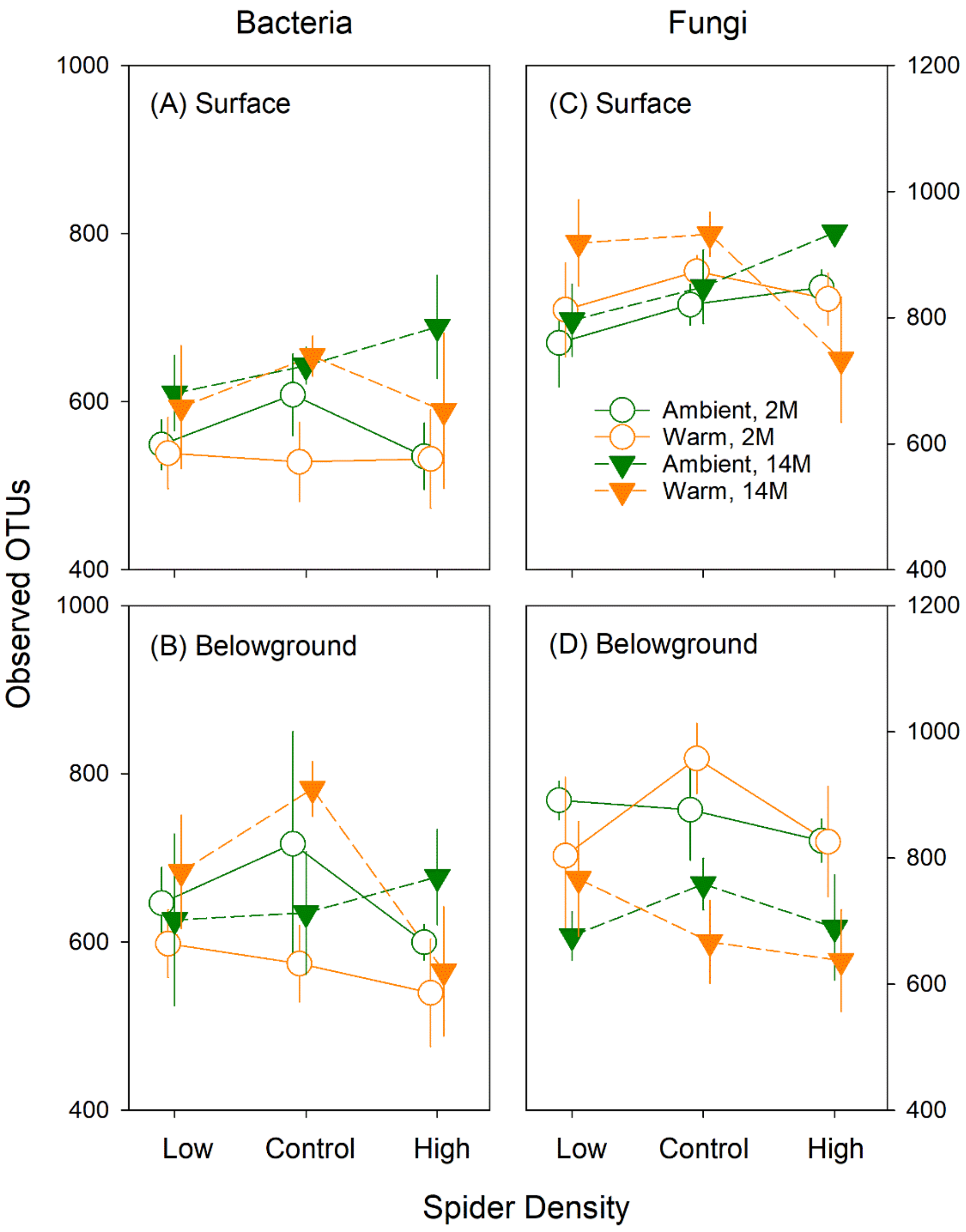

Fig. S2. Richness (observed OTUs) of bacterial and fungal communities in surface and belowground litter collected after two-month (2011) and 14-month (2012) incubations. Results of mixed-effect ANOVAs are shown in Table S2. 2M and 14M represent two-month and 14-month incubations, respectively. Error bars show standard errors.

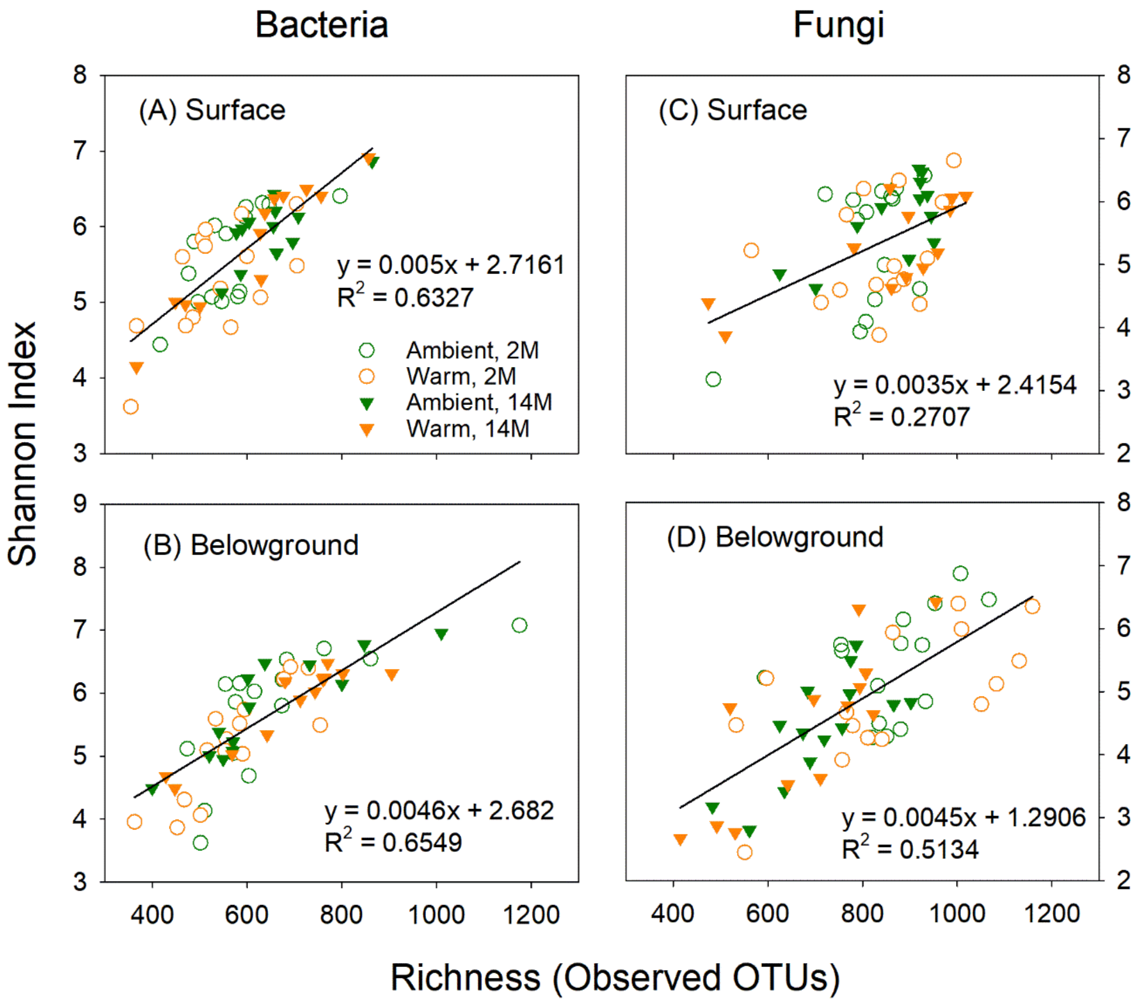

Fig. S3. Correlations between richness (i.e., observed OTUs) and Shannon Indices for bacterial and fungal communities in litter assessed for each soil profile.

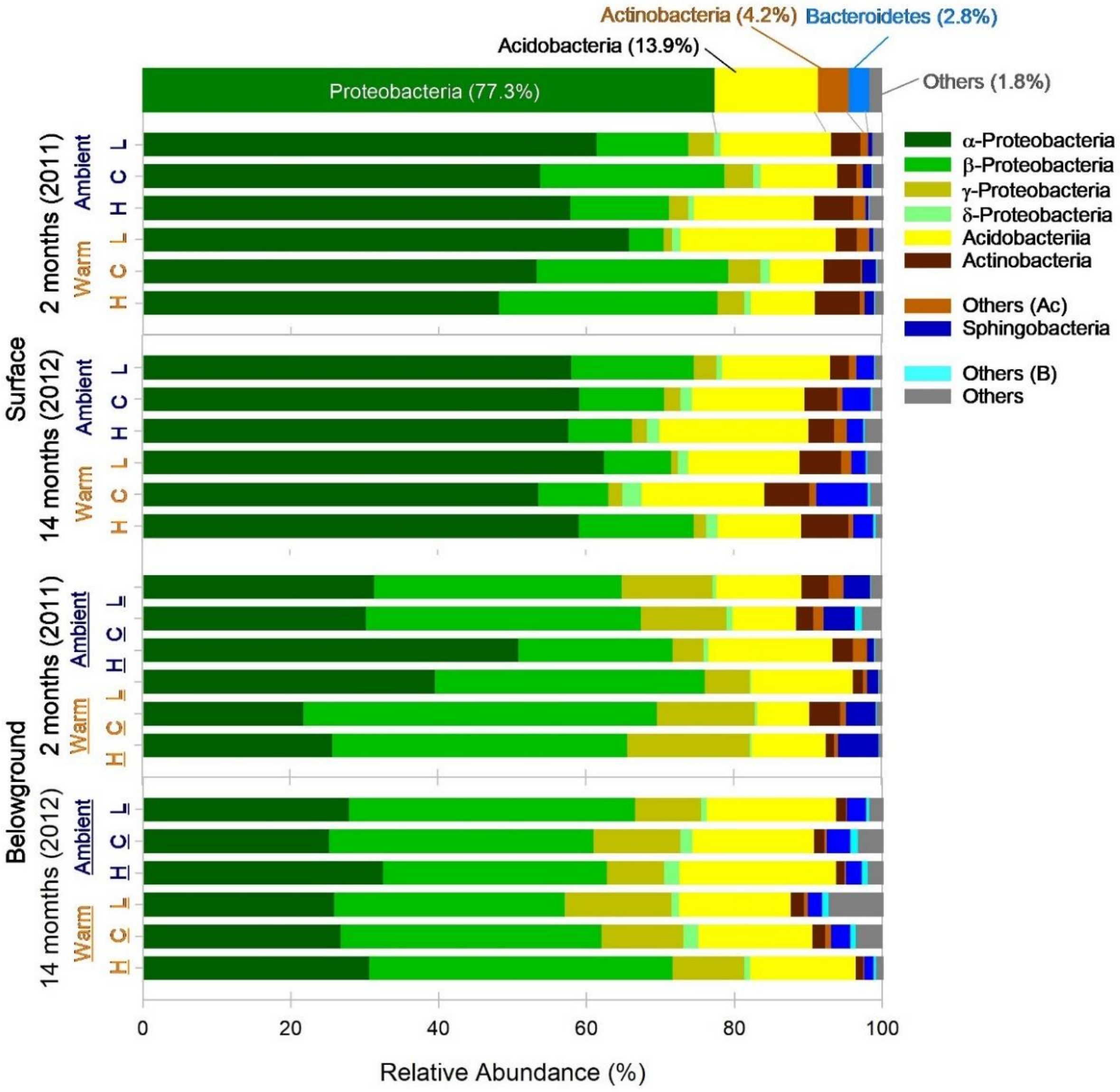

Fig. S4. Mean relative abundances of bacteria at the class level in surface and belowground litter collected in 2011 and 2012. Others (Ac) and (B) represent low abundance OTUs belonging to class Actionabacteria and Bacteroidetes, respectively. Others represent OTTs in phyla of low relative abundance other than the four major phyla.

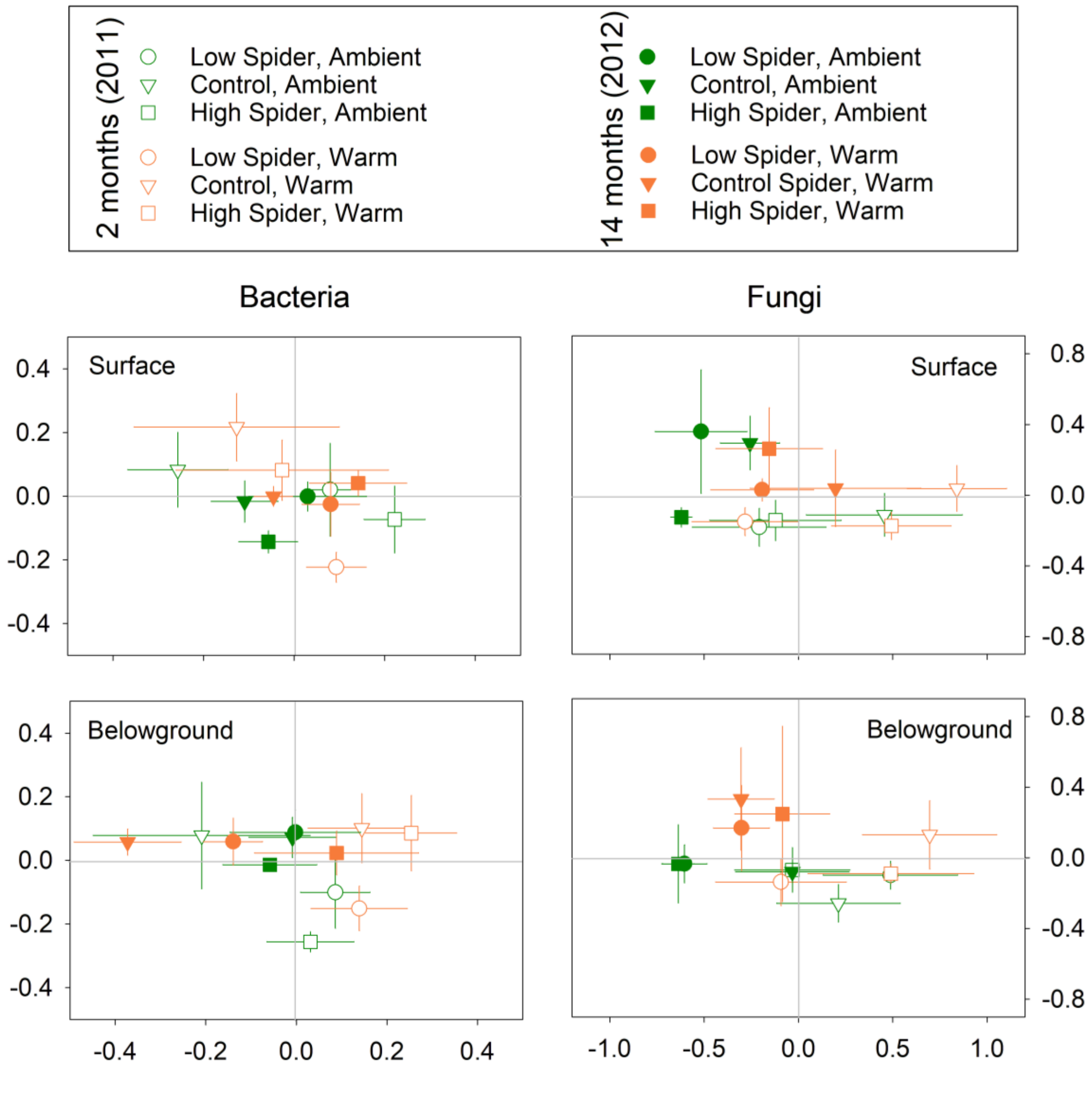

Fig. S5. Results of non-metric multi-dimensional scaling (NMDS) for bacterial and fungal communities at the class level in surface and belowground litter collected after two-month (2011) and 14-month (2012) incubations. NMDS was conducted for each microbial group and soil profile. Results of PERMANOVA are shown in Table S2. Error bars show standard errors.

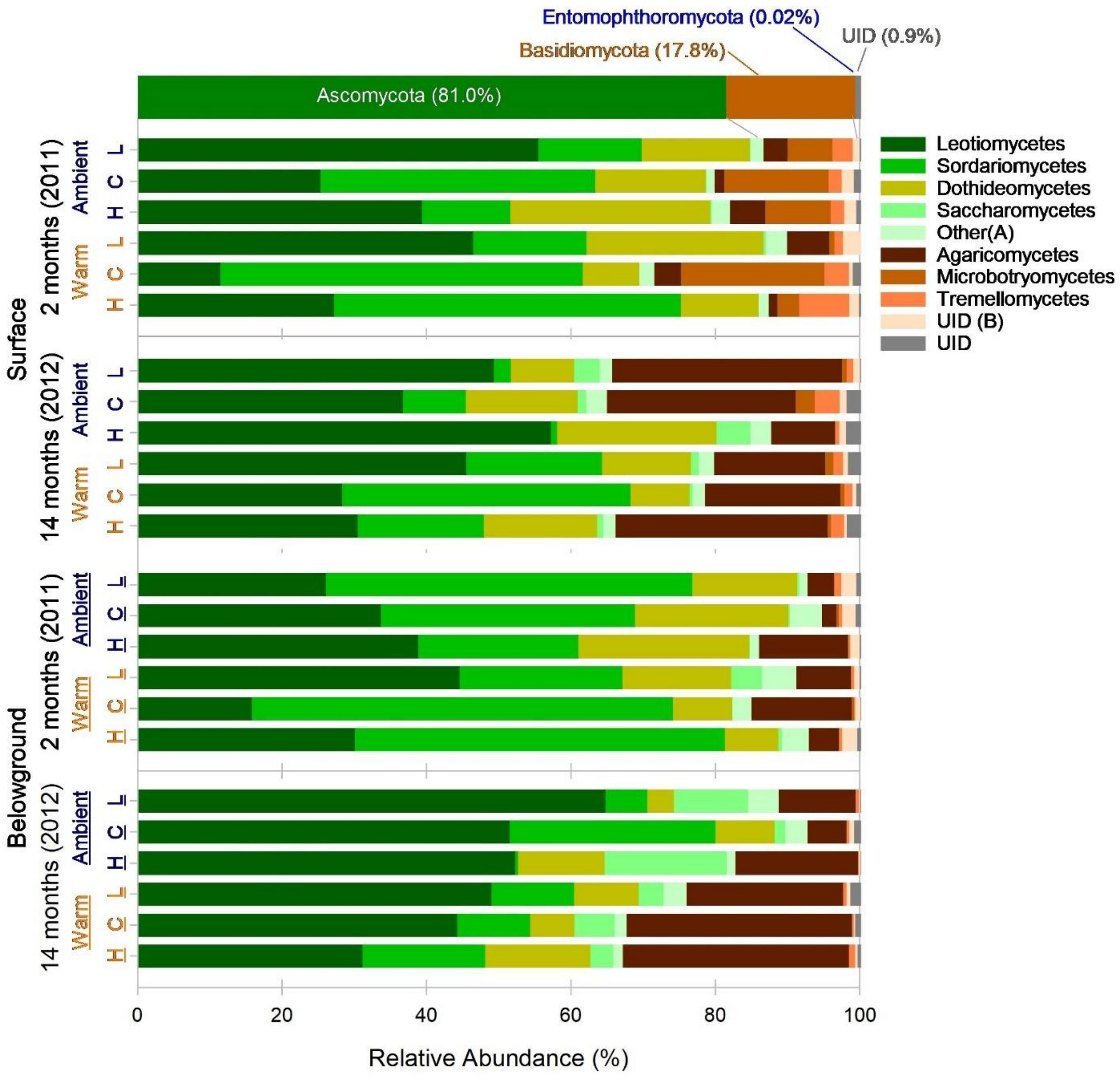

Fig. S6. Mean relative abundances of fungi at the class level in surface and belowground litter collected in 2011 and 2012. Others (A) represents low abundance OTUs belonging to class Ascomycota. UID (B) represents OUTs belonging to Basidiomycota and unidentified at the class level. UID represents OTUs unidentified at the phylum level.

Table S1. Results of mixed-effect ANOVAs for three litter characteristics (mass remaining and C:N ratio) analyzed by each profile and incubation time.

Time	Predictive variables	Mass Remaining		C:N Ratio	
		Surface	Belowground	Surface	Belowground
2 Months (2011)	Temp	$F_{1,20}$ = 1.05<br>*P* = 0.317	$F_{1,19}$ = 1.09<br>*P* = 0.310	$F_{1,20}$ = 1.26<br>*P* = 0.276	$F_{1,19}$ = 0.93<br>*P* = 0.347
	Spider	$F_{1,20}$ = 1.55<br>*P* = 0.236	$F_{1,19}$ = *1.52*<br>*P = 0.244*	$F_{1,20}$ = 4.27<br>***P* = 0.029**	$F_{1,19}$ = 0.14<br>*P* = 0.87
	Spider×Temp	$F_{1,20}$ = 2.93<br>*P* = 0.076	$F_{1,19}$ = 0.29<br>*P* = 0.755	$F_{1,20}$ = 1.98<br>*P* = 0.165	$F_{1,19}$ = 0.54<br>*P* = 0.594
14 Months (2012)	Temp	$F_{1,20}$ < 0.01<br>*P* = 0.987	$F_{1,20}$ = 1.93<br>*P* = 0.180	$F_{1,20}$ = 0.07<br>*P* = 0.793	$F_{1,20}$ = 0.61<br>*P* = 0.442
	Spider	$F_{1,20}$ = 1.42<br>*P* = 0.264	$F_{1,20}$ = 3.08<br>*P* = 0.068	$F_{1,20}$ = 1.01<br>*P* = 0.381	$F_{1,20}$ = 1.2<br>*P* = 0.322
	Spider×Temp	$F_{1,20}$ = 0.86<br>*P* = 0.437	$F_{1,20}$ = 8.66<br>***P* = 0.002**	$F_{1,20}$ = 0.32<br>*P* = 0.730	$F_{1,20}$ = 4.86<br>***P* = 0.019**

Significant effects (i.e., *P* < 0.05) are in bold.

Table S2. Results of mixed-effect ANOVAs for Richness (Observed OTUs).

Predictive variables	Bacteria		Fungi	
	Surface	Belowground	Surface	Belowground
Time	$F_{1,38}$ = 7.32<br>**P = 0.010**	$F_{1,39}$ = 1.58<br>P = 0.216	$F_{1,38}$ = 0.90<br>P = 0.348	$F_{1,41}$ = 15.8<br>**P < 0.001**
Temp	$F_{1,38}$ = 1.27<br>P = 0.266	$F_{1,39}$ = 0.45<br>P = 0.509	$F_{1,38}$ = 0.05<br>P = 0.820	$F_{1,41}$ = 0.06<br>P = 0.803
Spider	$F_{2,38}$ = 0.39<br>P = 0.680	$F_{2,39}$ = 1.49<br>P = 0.237	$F_{2,38}$ = 0.81<br>P = 0.453	$F_{2,41}$ = 0.95<br>P = 0.395
Time×Temp	$F_{1,38}$ = 0.01<br>P = 0.932	$F_{1,39}$ = 2.16<br>P = 0.150	$F_{1,38}$ = 0.48<br>P = 0.494	$F_{1,41}$ = 0.04<br>P = 0.842
Time×Spider	$F_{2,38}$ = 0.17<br>P = 0.846	$F_{2,39}$ = 0.08<br>P = 0.927	$F_{2,38}$ = 0.54<br>P = 0.589	$F_{2,41}$ = 0.28<br>P = 0.760
Temp×Spider	$F_{2,38}$ = 0.11<br>P = 0.895	$F_{2,39}$ = 0.40<br>P = 0.671	$F_{2,38}$ = 3.33<br>**P = 0.047**	$F_{2,41}$ = 0.02<br>P = 0.976
Time×Temp×Spider	$F_{2,38}$ = 0.84<br>P = 0.441	$F_{2,39}$ = 1.46<br>P = 0.244	$F_{2,38}$ = 1.48<br>P = 0.241	$F_{2,41}$ = 1.62<br>P = 0.211

Significant effects (i.e., P < 0.05) are in bold.

Table S3. Results of PERMANOVA for NMDS scores at the Class level.

Predictive variables	Bacteria		Fungi	
	Surface	Belowground	Surface	Belowground
Time	$F_{1,53}$ = 0.19<br>P = 0.811<br>R^2 = 0.00	$F_{1,54}$ = 3.85<br>**P = 0.035**<br>R^2 = 0.06	$F_{1,53}$ = 6.52<br>**P = 0.003**<br>R^2 = 0.10	$F_{1,56}$ = 11.26<br>**P = 0.001**<br>R^2 = 0.16
Temp	$F_{1,53}$ = 0.31<br>P = 0.749<br>R^2 = 0.01	$F_{1,54}$ = 0.63<br>P = 0.545<br>R^2 = 0.01	$F_{1,53}$ = 2.97<br>P = 0.067<br>R^2 = 0.05	$F_{1,56}$ = 0.08<br>P = 0.909<br>R^2 = 0.00
Spider	$F_{2,53}$ = 3.40<br>**P = 0.016**<br>R^2 = 0.12	$F_{2,54}$ = 2.33<br>P = 0.071<br>R^2 = 0.08	$F_{2,53}$ = 3.64<br>**P = 0.010**<br>R^2 = 0.12	$F_{2,56}$ = 1.48<br>P = 0.233<br>R^2 = 0.04
Time×Temp	$F_{1,53}$ = 0.67<br>P = 0.528<br>R^2 = 0.01	$F_{1,54}$ = 3.53<br>**P = 0.036**<br>R^2 = 0.06	$F_{1,53}$ = 0.13<br>P = 0.838<br>R^2 = 0.00	$F_{1,56}$ = 0.84<br>P = 0.427<br>R^2 = 0.01
Time×Spider	$F_{2,53}$ = 0.90<br>P = 0.454<br>R^2 = 0.03	$F_{2,54}$ = 0.52<br>P = 0.727<br>R^2 = 0.02	$F_{2,53}$ = 0.72<br>P = 0.510<br>R^2 = 0.02	$F_{2,56}$ = 0.41<br>P = 0.782<br>R^2 = 0.01
Temp×Spider	$F_{2,53}$ = 1.27<br>P = 0.306<br>R^2 = 0.05	$F_{2,54}$ = 1.13<br>P = 0.342<br>R^2 = 0.04	$F_{2,53}$ = 0.61<br>P = 0.580<br>R^2 = 0.02	$F_{2,56}$ = 3.27<br>**P = 0.037**<br>R^2 = 0.09
Time×Temp×Spider	$F_{2,53}$ = 1.02<br>P = 0.429<br>R^2 = 0.04	$F_{2,54}$ = 1.38<br>P = 0.251<br>R^2 = 0.04	$F_{2,53}$ = 0.59<br>P = 0.620<br>R^2 = 0.02	$F_{2,56}$ = 1.26<br>P = 0.287<br>R^2 = 0.04

Significant effects (i.e., $P < 0.05$) are in bold.
